# Novel Application of Oral Postbiotics for Skin Condition in Healthy Middle‐Aged Women: A Randomized, Double‐Blind, and Placebo‐Controlled Study

**DOI:** 10.1111/jocd.70617

**Published:** 2025-12-18

**Authors:** Jinko Sawashita, Shinichi Honda, Yuji Tominaga, Namiko Tomatsu, Jordi Espadaler‐Mazo, Takahiro Ueda

**Affiliations:** ^1^ Scientific Affairs Team, Planning Group, Supplement Strategic Unit, Pharma & Supplemental Nutrition Solutions Vehicle Kaneka Corporation Osaka Japan; ^2^ Probiotics Team, Supplement & Probiotics Research Laboratories Kaneka Corporation Hyogo Japan; ^3^ Scientific Affairs Team, Planning Group, Supplement Strategic Unit, Pharma & Supplemental Nutrition Solutions Vehicle Kaneka Corporation Tokyo Japan; ^4^ Department of Research & Development AB‐Biotics SA (Kaneka Group) Barcelona Spain; ^5^ Probiotics Business Group, Supplement Strategic Unit, Pharma & Supplemental Nutrition Solutions Vehicle Kaneka Corporation Tokyo Japan

**Keywords:** dry skin, heathy women, *Latilactobacillus sakei*, middle‐aged, postbiotics

## Abstract

**Background and Purpose:**

*Latilactobacillus sakei* KABP‐065 derived from pickled vegetables of East Asia can improve skin condition in patients with atopic dermatitis through a mechanism that suppresses immune hyperactivity. Here we investigated whether KABP‐065 postbiotics can improve the facial skin condition of healthy middle‐aged women.

**Methods:**

Japanese women (*n* = 60) aged ≥ 30 to < 50 years who met inclusion criteria were randomly assigned to postbiotic or placebo groups. The participants consumed capsules containing either heat‐treated KABP‐065 (1 × 10^10^ cells/day) or placebo for 8 weeks. The effect of the postbiotic on skin condition parameters was assessed on the skin of the cheeks before the intervention, and at 5 and 8 weeks after the intervention. Subjective evaluations of skin conditions and mental and gastrointestinal symptoms, as well as safety evaluations, were also conducted.

**Results:**

Cheek skin moisture and transepidermal water loss improved for both groups after the intervention. The postbiotic group had significantly improved skin elasticity relative to the placebo group. A stratified analysis of participants in their 40s showed that, compared to the placebo group, the postbiotic group had a greater improvement in skin elasticity and a significantly greater improvement in cheek moisture. No exacerbation of gastrointestinal symptoms was seen with postbiotic supplementation, and the frequency of adverse events was similar between the postbiotic and placebo groups.

**Conclusion:**

KABP‐065 postbiotics could be effective for improving the skin condition of healthy women aged ≥ 30 to < 50 years, especially those in their 40s.

**Abbreviations**
AD: atopic dermatitis
CRO: contract research organization
FAS: full analysis set
Ig: immunoglobulin
LMM: linear mixed‐effects model
PPS: per‐protocol set
TEWL: transepidermal water loss
VAS: visual analog scales

## 1 Introduction

Skin aging can be caused by external physical and chemical factors such as sunlight and climate/weather conditions, as well as by internal factors such as age‐related decreases and changes in the interactions of skin components such as elastin, ceramide, and hyaluronic acid, as well as diminished barrier function due to immune dysregulation [1]. It is also associated with thinning of the epidermis, delayed turnover, reduced sebum secretion, reduced stratum corneum moisture, and flattening of the dermal‐epidermal junction, which can lead to noticeable changes in skin appearance and the occurrence of skin diseases [2, 3, 4, 5]. Several strategies have been proposed to address external and/or internal factors of skin aging, such as moisturizing with products containing *Aloe vera* [6] or supplementation with the antioxidant coenzyme Q10 that can help ameliorate skin cell/component damage caused by reactive oxygen species [7]. Meanwhile, lactic acid bacteria can modulate immune activity, and there is growing evidence for the effectiveness of treatments containing these bacterial strains [8]. In particular, probiotics can modify components of the intestinal microbiome, generate antimicrobial substances, and maintain immune system function [9]. Recent evidence suggests that probiotics can protect the skin from adverse internal and/or external stimuli [10].

The gut‐brain‐skin axis is a bidirectional network mediated by the gut microbiota, neuroendocrine system, and immune system. Changes in the intestinal environment and psychological stress are thought to affect skin inflammation and barrier function, and these changes could contribute to an increased understanding of and new treatments for conditions like acne, atopic dermatitis (AD), and psoriasis [11]. Furthermore, Tsilingiri and Rescignos defined soluble factors such as butyric acid and acetic acid contained in bacteria as “postbiotics”, and hypothesized that several biological responses in hosts who consumed supplements containing non‐viable bacteria are due to postbiotic function [12]. For example, Horii et al. found that heat‐treated *Lactobacillus brevis* SBC8803 suppressed cutaneous arterial sympathetic nerve activity, increased skin blood flow, and decreased transepidermal water loss (TEWL) [13]. This finding suggests that the mechanism of action for these beneficial effects involves 5‐HT_3_ receptors in the serotonin nervous system and vagus nerves. Moreover, several studies suggested that non‐viable lactic acid bacteria retain immunomodulatory properties [14, 15]. Proper control of the immune system is reported to be essential not only for pathological skin conditions but also for maintaining healthy skin barrier function [16].

A general problem with probiotics is loss of stability during storage [17], particularly when probiotics are present in a closed space like a food capsule that lacks nutrients and an environment that supports viability. Indeed, storage stability has been shown to be an issue for live *Latilactobacillus sakei* [18], and studies are currently underway to improve its stability. Sionek and Gantner suggested that the shelf life of probiotics is limited by the stability of live bacteria [19], making stability an important consideration for manufacture of formulations containing probiotics. In contrast, non‐viable strains do not have the limitations of viable probiotics and can be stored for longer periods, making non‐viable strains potentially safer and more cost‐effective compared to viable strains.

*L. sakei* KABP‐065, which is derived from traditional pickled vegetables that are frequently consumed in East Asia, has been suggested to have beneficial immunomodulatory and/or immunosuppressive effects by promoting production of immunomodulatory substances [20, 21, 22]. This lactic acid bacteria strain has also been shown to be effective for treating AD, a skin disease characterized by dryness, scaling, pruritic lesions, and lichenification [23, 24]. Like other probiotics, both live and non‐viable *L. sakei* KABP‐065 were shown to significantly improve skin condition by reducing levels of serum immunoglobulin (Ig) E and cutaneous T cell‐attracting chemokines, both in AD‐prone model mice [25] and in a clinical trial [26]. For the present study, we hypothesized that non‐viable *L. sakei* KABP‐065 would also have beneficial effects for healthy skin. We carried out a clinical trial to examine whether KABP‐065 postbiotics have beneficial effects for the skin of healthy adult women who consumed oral supplements containing KABP‐065 daily for 8 weeks.

## 2 Materials and Methods

### 2.1 Ethics

This clinical study protocol was approved by the ethics committees named at the Clinical Research Review Center (Tokyo, Japan; January 11, 2023; approval No. CrrC23‐02). This study was also registered at the University Hospital Medical Information Network Clinical Trials Registry in Japan (registration No. UMIN000050034).

All volunteers provided written informed consent to participate. The study was performed in accordance with the Declaration of Helsinki (adopted in 1964 and revised in October 2013), the Ethical Guidelines for Medical and Health Research Involving Human Subjects in Japan (Notification No. 3 issued in 2014 by the Ministry of Education, Culture, Sports, Science and Technology and the Ministry of Health, Labour and Welfare in Japan), and the Act on the Protection of Personal Information (Act No. 57 issued May 30, 2003 in Japan)Study Design and Protocol.

### 2.2 Study Design and Protocol

This study involved a randomized, double‐blind, placebo‐controlled, parallel‐group comparison carried out at a single clinical institute, the Ebisu Skin Research Center (Tokyo, Japan), between January 2023 and May 2023 (the intervention period was from February 2023 to April 2023). Based on scientific reviews by Julious [27] and Hertzog [28], we determined that 60 participants were required for this study, which is the first trial involving healthy individuals and the tested postbiotic food. The study food allocation manager (Tsurumi University, Kanagawa, Japan), which was commissioned by a contract research organization (CRO), assigned 60 participants to receive either postbiotic or placebo capsules using a stratified block randomization method that considered the water content on the stratum corneum of the cheek skin in participants. The allocation manager maintained the study allocation list and all parties remained blinded until study completion.

### 2.3 Participants

We enrolled healthy volunteers who met the following inclusion criteria: (1) Japanese women aged ≥ 30 to < 50 years at the time of informed consent; (2) those who routinely expressed concern about dry skin, but who were judged not to have any dermatological disorders after review by a medical doctor; and (3) those who received a sufficient explanation of the purpose and content of the study and expressed a full understanding of the study purpose and content before providing consent in writing of their intention to participate in the study voluntarily. Participants were excluded who: (1) regularly used medicines, foods, or supplements that affected skin condition (other *lactobacillus* foods that were reported before participation and continued to be taken during the participation period were not applicable); (2) had skin disease symptoms such as atopic dermatitis, or scars/inflammation at the test sites on the face; (3) had severe hay fever; (4) had allergies to components of the test food; (5) had hepatic, renal, gastrointestinal, cardiac, respiratory or endocrine diseases or had other metabolic systemic disorders; (6) had a history of cosmetic or medical treatments, underwent hormone replacement therapy within the past 6 months or intended to undergo such treatments during the study period; (7) had digestive diseases or a history of surgery of the digestive tract; (8) were pregnant, lactating, or intending to become pregnant; (9) planned to undergo tanning treatments or visit areas with a possibility of exposure to strong sunlight such as the seashore, the mountains, outdoor amusement parks, or sporting venues during the study period; (10) drank ≥ 20 g/day pure alcohol for ≥ 4 days per week or who could not abstain from alcohol for 2 days before each examination; (11) were shift workers, late night workers, or planned to travel abroad; (12) could not avoid changing or adding skin care products (even when visiting the inspection site); (13) could not avoid shaving the skin for 7 days before each examination; (14) participated in other studies within 1 month of the date of informed consent, or who planned to participate in other studies during the study period; or (15) were deemed inappropriate for inclusion by the corresponding investigator who has a medical license. Participants were requested not to change their lifestyle or eating and drinking habits during the intervention period after preregistration. In particular, some participants were taking foods that included other *Lactobacillus* bacteria (e.g., yogurt and Japanese pickled vegetables) at preregistration. These participants were urged to continue taking the same bacteria without changing the frequency or amount. For 1 week before the intervention and during the intervention period, all subjects used lifestyle‐related diaries to answer questions concerning test food intake, physical condition, dietary changes, needed medical treatment, health/supplement foods consumed, consumption of other foods or undertaking actions (behavior) that may affect the study results, treatments involving cosmetics, the amount of alcohol consumed and the amount/type of exercise undertaken.

### 2.4 Preparation of the Test Foods and Intervention

Heat‐treated *Latilactobacillus sakei* KABP‐065 (KCTC 10755BP) was obtained from the Probionic Corporation ( *Lactobacillus sakei* Probio65, Jeonbuk State, South Korea). Sunsho Pharmaceutical Co. Ltd. (Shizuoka, Japan) was used to manufacture postbiotic capsules containing 1 × 10^10^ *L. sakei* KABP‐065 cells according to Japanese food processing standards. In addition to *L. sakei* KABP‐065, the postbiotic capsules contained starch, calcium stearate, hydroxypropyl methylcellulose, and titanium dioxide. Placebo capsules manufactured by the same company were indistinguishable in form, color, and taste from the postbiotic capsule and contained starch substituted for *L. sakei* KABP‐065. Both capsule types were then placed under control of the CRO (KSO Co. Ltd., Tokyo, Japan). All capsules were stored away from sunlight at ≤ 25°C. Each participant was instructed to take 1 capsule after a meal (breakfast was recommended) for 8 weeks.

### 2.5 Efficacy and Safety Assessment

During the study period, participants visited the clinical site where they washed their face with water and their usual face soap. The participants stayed under conditions with controlled temperature at 21°C ± 1°C and relative humidity of 50% ± 5% for 15 min before facial parameters were assessed.

The primary efficacy endpoints were essential parameters for cheek skin condition and included: skin moisture (stratum corneum water content) measured with a Corneometer CM825 (Courage+Khazaka Electronic GmbH, Germany); TEWL measured by a Tewameter Hex (Courage+Khazaka Electronic GmbH, Germany); and skin elasticity measured with a Cutometer dual MPA580 (Courage+Khazaka Electronic GmbH, Germany) [29]. As secondary efficacy endpoints, an objective evaluation of erythema and melanin levels at the intersection of the right eye corner and nose was carried out using a Mexameter MX‐18 instrument (Courage+Khazaka Electronic GmbH, Germany), and a subjective evaluation of parameters such as dryness, itchiness, dullness, and pore condition from the nose to cheek was assessed using a visual analog scales (VAS) questionnaire. The mental and gastrointestinal conditions of the participants were evaluated using a VAS questionnaire and the Izumo scale score [30], respectively, as additional subjective parameters.

For safety evaluation, the participants' height, body weight, and systolic and diastolic blood pressure were measured. Blood, hematology, and urine analyses were also performed. Biochemical parameters of blood and urine samples were measured at the clinical center according to standard procedures recommended by the Japanese Ministry of Health, Labor and Welfare at the time of health examination. A physician assessed the results of blood biochemical and hematologic analyses and urinalysis as well as adverse events based on participant communication. Participants' faces were also examined by a dermatologist. The dermatologist graded the individual's skin conditions according to the inspection site's manual. For example, the degree of skin dryness was determined using standard photographs. Daily diaries in which participants recorded intake of the test food, presence/absence of medical treatment, and lifestyle‐related changes were also used to evaluate study compliance.

### 2.6 Statistical Analysis

**FIGURE 1:**
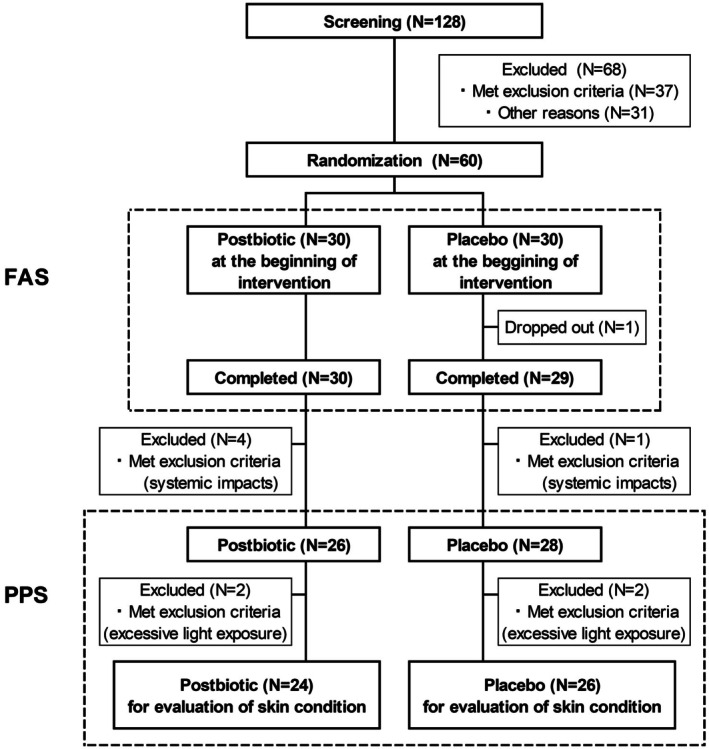
Flowchart of study enrollment. A total of 60 participants were enrolled after initial screening. The postbiotic and placebo groups had 30 and 29 participants, respectively, who completed the 8‐week study period and these individuals were included in the full analysis set (FAS). Two participants were excluded from each group due to excessive light exposure, so the postbiotic and placebo per‐protocol (PPS) set included 26 and 28 participants, respectively.

We used the SPSS Statistics 28 software package (IBM). Analyses of efficacy and safety were performed using a per‐protocol set (PPS) and full analysis set (FAS) population, respectively (Figure 1). Statistical analyses for parametric parameters including skin moisture, TEWL, and skin elasticity, as well as physical and vital signs and blood biochemistry, were performed using Student's unpaired *t*‐test or Welch's *t*‐test for intergroup comparisons; Dunnett's test or paired *t*‐test was used for intragroup comparisons. Other analyses for non‐parametric parameters were conducted using a Mann–Whitney *U‐*test for intergroup comparisons and Wilcoxon signed rank exact test for intragroup comparisons. Evaluation of adverse events was performed using Fisher's exact test. Two‐sided *p* values < 0.05 were considered statistically significant.

## 3 Results

### 3.1 Participants

After screening, 60 participants were enrolled and randomized into postbiotic or placebo groups that included 30 and 29 participants, respectively, who completed the study and underwent safety evaluations (Figure 1). Consumption of the postbiotic or placebo capsules was 100%. At the end of the 8‐week study period, 4 and 1 participants in the postbiotic and placebo groups, respectively, were excluded from all efficacy analyses based on a physician's judgment of serious non‐compliance that would have systemic impacts including disruptive lifestyle changes, changes in dietary habits, and prolonged use of pharmaceuticals. There were no significant differences in any baseline characteristic between the postbiotic and placebo groups (Tables 1 and 2). An additional 2 participants from each group were excluded from the analyses of skin‐related parameters due to excessive light exposure events, such as visiting an outdoor amusement park and/or having prolonged sun exposure (Figure 1). The efficacy analyses of skin condition included 24 participants from the postbiotic group and 26 participants from the placebo group.

**TABLE 1:** Baseline data for study participants.

Parameter	Postbiotic (*N* = 26)	Placebo (*N* = 28)	*p*
Age (year)	42.0 ± 5.3	41.2 ± 6.4	0.612
Height (cm)	156.9 ± 5.3	156.6 ± 6.0	0.819
Body weight (kg)	54.0 ± 10.7	51.5 ± 7.1	0.310
Systolic blood pressure (mmHg)	112.0 ± 11.2	113.6 ± 15.6	0.675
Diastolic blood pressure (mmHg)	70.5 ± 11.2	71.6 ± 9.5	0.716
Moisture on cheek skin (a.u.)	31.7 ± 6.1	31.1 ± 6.1	0.706

*Note:* Values are mean ± standard deviations. *p* values were derived from comparisons between the postbiotic and placebo groups using unpaired Student's or Welch's *t*‐test. There was no significant difference between the groups for any parameter.

Abbreviation: a.u., arbitrary unit.

**TABLE 2:** Baseline blood and urine data.

Parameter		Postbiotic (*N* = 26)	Placebo (*N* = 28)	*p*
Blood				
White blood cells (/μL)		6015 ± 1593	6132 ± 1672	0.794
Red blood cells (×10^4^/μL)		431.8 ± 32.7	435.1 ± 22.8	0.668
Hemoglobin (g/dL)		13.0 ± 0.9	12.9 ± 0.8	0.486
Hematocrit (%)		40.6 ± 2.8	40.1 ± 2.5	0.456
Platelet (×10^4^/μL)		28.9 ± 7.5	27.4 ± 5.3	0.409
MCV (fL)		94.2 ± 5.2	92.1 ± 4.4	0.114
MCH (pg)		30.3 ± 2.1	29.6 ± 1.6	0.198
MCHC (%)		32.2 ± 0.8	32.2 ± 0.8	0.950
Total protein (g/dL)		7.3 ± 0.4	7.2 ± 0.3	0.803
Albumin (g/dL)		4.4 ± 0.2	4.4 ± 0.2	0.336
AST (U/L)		18.7 ± 5.0	18.0 ± 4.2	0.544
ALT (U/L)		14.3 ± 6.7	14.6 ± 6.3	0.850
LDH (U/L)		169.4 ± 26.0	157.6 ± 24.8	0.095
Total bilirubin (mg/dL)		0.7 ± 0.2	0.7 ± 0.2	0.690
ALP (U/L)		55.8 ± 14.9	59.9 ± 14.3	0.309
γ‐GTP (U/L)		18.8 ± 13.6	15.0 ± 5.1	0.192
Urea nitrogen (mg/dL)		11.8 ± 2.5	13.0 ± 4.0	0.185
Creatinine (mg/dL)		0.62 ± 0.09	0.59 ± 0.07	0.142
Uric acid (mg/dL)		4.1 ± 0.7	3.8 ± 0.8	0.141
Sodium (mEq/L)		140.6 ± 2.1	140.0 ± 1.5	0.217
Chloride (mEq/L)		104.1 ± 2.0	103.6 ± 1.7	0.393
Potassium (mEq/L)		4.3 ± 0.3	4.3 ± 0.4	0.677
Calcium (mg/dL)		9.4 ± 0.3	9.4 ± 0.3	0.419
Total‐Cho (mg/dL)		205.3 ± 38.6	201.7 ± 37.3	0.727
LDL‐Cho (mg/dL)		114.3 ± 30.7	112.2 ± 28.7	0.790
HDL‐Cho (mg/dL)		78.3 ± 22.6	76.4 ± 17.9	0.735
Triglyceride (mg/dL)		57.4 ± 26.5	66.8 ± 35.4	0.281
Fasting glucose (mg/dL)		83.2 ± 8.4	81.3 ± 5.4	0.333
Hemoglobin A1c (%)		5.2 ± 0.3	5.2 ± 0.2	0.787
Urine				
pH		6.0 ± 0.4	6.1 ± 0.6	0.370
Gravity		1.023 ± 0.006	1.020 ± 0.008	0.214
Protein, qualitative (numbers)	−	23	25	0.924
	±	3	3	
	1+	0	0	
	2+	0	0	
	3+	0	0	
Glucose, qualitative (numbers)	−	26	28	1.000
	±	0	0	
	1+	0	0	
	2+	0	0	
	3+	0	0	
Urobilinogen, qualitative (numbers)	−	0	0	0.299
	±	25	28	
	1+	1	0	
Bilirubin, qualitative (numbers)	−	26	28	1.000
	±	0	0	
	1+	0	0	
	2+	0	0	
	3+	0	0	
Ketone, qualitative (numbers)	−	25	28	0.299
	±	0	0	
	1+	1	0	
	2+	0	0	
	3+	0	0	
Blood, quantitative (numbers)	−	23	21	0.210
	±	1	2	
	1+	1	2	
	2+	0	2	
	3+	1	1	

*Note:* Values are mean ± standard deviations or numbers of subjects. *p* values were derived from comparisons between the postbiotic and placebo groups using unpaired Student's or Welch's *t*‐test (for quantitative parameters) or Wilcoxon's rank sum exact test (for qualitative parameters). There was no significant difference between the groups for any parameter.

Abbreviations: ALP, alkaline phosphatase; ALT, alanine aminotransferase; AST, aspartate aminotransferase; Cho, cholesterol; LDH, lactate dehydrogenase; MCH, mean corpuscular hemoglobin; MCHC, mean corpuscular hemoglobin‐concentration; MCV, mean corpuscular volume; γ‐GPT, γ‐glutamic transpeptidase.

### 3.2 Primary Efficacy Endpoints

**FIGURE 2:**
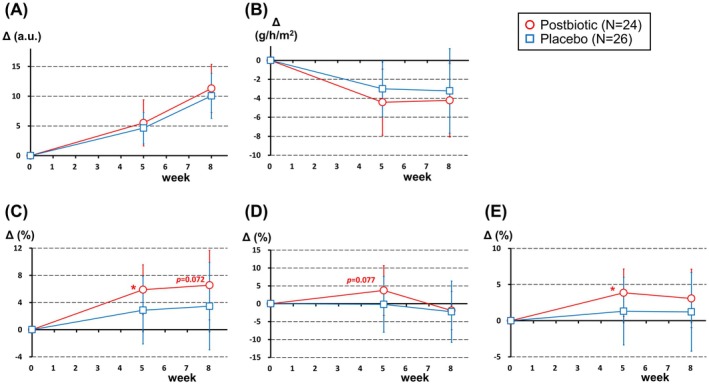
Effect of postbiotics on changes in three skin parameters used as primary endpoints. Data for groups given the postbiotic and placebo are shown in red and blue, respectively. Open symbols correspond to mean values and error bars indicate standard deviations. Delta values at 5 and 8 weeks after intervention are represented as the change in value relative to the baseline (week 0). (A) Stratum corneum water content, (B) transepidermal water loss (TEWL), (C–E) skin elasticity. (C) Skin recovery rate (R2); (D) net elasticity (R5); (E) skin firmness (R7). *p < 0.05 and other *p* values were derived from between‐group comparisons.

In an objective, intragroup comparison of parameters before and after the intervention, participants in both the postbiotic and placebo groups showed significant improvements in stratum corneum water content and TEWL of the cheek skin (Table 3). However, no significant differences were observed between the postbiotic and placebo groups in terms of the changes in these parameters from baseline to each intervention time point (Figure 2A,B). The R2 of skin elasticity, which reflects the skin recovery rate, increased over time in both groups (Table 3). In the postbiotic group, the change in the R2 value after 5 weeks of intervention was significantly larger (*p* = 0.022) and the change after 8 weeks of intervention tended to be larger (*p* = 0.072) relative to the placebo group (Figure 2C). R5 (net elasticity) and R7 (skin firmness) appeared to increase over time for the postbiotic group, but not for the placebo group (Table 3). The positive change in those parameters tended to be larger for the postbiotic group compared to the placebo group (*p* = 0.077 for R5 and *p* = 0.036 for R7 at 5 weeks after intervention) (Figure 2D,E).

**TABLE 3:** Objective evaluation of cheek skin parameters.

Parameter	Postbiotic (*N* = 24)	Placebo (*N* = 26)	*p*
Stratum corneum water content (a.u.)			
Baseline	32.4 ± 7.0	33.3 ± 6.1	0.647
5 weeks	37.9 ± 8.3^###^	37.9 ± 6.7^###^	0.992
8 weeks	43.7 ± 6.6^###^	43.3 ± 7.3^###^	0.836
TEWL (g/h/m^2^)			
Baseline	19.6 ± 4.7	20.2 ± 6.5	0.723
5 weeks	15.2 ± 3.6^###^	17.2 ± 4.7^###^	0.101
8 weeks	15.4 ± 3.9^###^	17.0 ± 5.6^###^	0.261
Elasticity (%)			
R2			
Baseline	58.8 ± 8.9	61.4 ± 7.7	0.281
5 weeks	64.7 ± 7.3^###^	64.2 ± 7.4^#^	0.832
8 weeks	65.4 ± 10.8^###^	64.8 ± 8.3^##^	0.842
R5			
Baseline	55.2 ± 9.2	56.8 ± 8.8	0.534
5 weeks	58.9 ± 8.0^#^	56.6 ± 8.9	0.349
8 weeks	53.3 ± 8.2	54.6 ± 8.5	0.598
R7			
Baseline	36.0 ± 6.6	38.3 ± 6.5	0.240
5 weeks	39.9 ± 5.9^###^	39.6 ± 7.1	0.859
8 weeks	39.1 ± 7.9^###^	39.5 ± 7.6	0.877
Erythema (a.u.)			
Baseline	256.0 ± 74.3	258.7 ± 68.6	0.895
5 weeks	218.5 ± 50.6^###^	238.2 ± 60.2^#^	0.219
8 weeks	206.5 ± 54.7^###^	230.7 ± 67.3^###^	0.172
Melanin (a.u.)			
Baseline	115.4 ± 31.1	125.3 ± 37.0	0.312
5 weeks	121.0 ± 27.7	127.5 ± 34.2	0.466
8 weeks	121.2 ± 26.4	130.0 ± 34.6	0.322

*Note:* Values are mean ± standard deviations. *p* values were derived from comparisons between the postbiotic and placebo groups (intergroups) using an unpaired *t*‐test. ^#^ *p* < 0.05, ^##^ *p* < 0.01, ^###^ *p* < 0.001 versus baseline within the same group using Dunnett's test.

Abbreviations: a.u., arbitrary unit; R2, skin recovery rate (total recovery amplitude/maximum amplitude by suction, Ua/Uf); R5, net elasticity (immediate refractive, Ur/Ue); R7, skin firmness (immediate recovery after relax/maximum amplitude by suction, Ur/Uf); TEWL, transepidermal water loss.

### 3.3 Secondary Efficacy Endpoints

For both the postbiotic and placebo groups, the amount of erythema in an objective analysis decreased significantly over the course of the intervention, but the amount of melanin did not change (Table 3). There were also no significant differences between the two groups in terms of erythema or melanin.

As subjective evaluation indicators, the VAS scores for dullness of the skin on the face and pores on the nose and cheeks were significantly reduced by week 8 of the postbiotic intervention compared to the placebo (*p* = 0.048 and 0.045, respectively) (Table 4). In subjective evaluation of other parameters related to the face as well as in an evaluation of psychological issues, some parameters showed improvement after the intervention in both groups, but none differed between the postbiotic and placebo groups. No participant in either group had any gastrointestinal problems before the start of the intervention, and the post‐intervention survey showed no exacerbations in either group (Table 5). There were also no complaints of any unpleasant gastrointestinal symptoms.

**TABLE 4:** Subjective assessment parameters for skin condition and evaluation of psychological issues associated with daily life.

Parameter	Postbiotic (*N* = 24)	Placebo (*N* = 26)	*p*
Skin dryness (%)			
Baseline	73.4 ± 11.5	70.5 ± 18.2	0.506
5 weeks	52.9 ± 15.2^###^	49.2 ± 21.5^###^	0.482
8 weeks	47.5 ± 20.7^###^	49.7 ± 20.7^###^	0.711
Δ5	−20.5 ± 18.2	−21.3 ± 17.3	0.867
Δ8	−25.9 ± 21.7	−20.8 ± 17.4	0.361
Skin elasticity (%)			
Baseline	73.1 ± 12.5	72.8 ± 16.3	0.946
5 weeks	59.1 ± 14.0^###^	59.4 ± 17.0^###^	0.946
8 weeks	59.5 ± 15.0^###^	62.0 ± 17.3^##^	0.590
Δ5	−14.0 ± 18.5	−13.4 ± 15.7	0.906
Δ8	−13.6 ± 18.2	−10.8 ± 18.0	0.591
Skin itchiness (%)			
Baseline	25.6 ± 26.2	34.0 ± 29.4	0.292
5 weeks	19.8 ± 21.1	21.1 ± 17.8^##^	0.811
8 weeks	23.8 ± 22.0	20.2 ± 22.0^##^	0.574
Δ5	−5.8 ± 20.7	−12.9 ± 21.6	0.242
Δ8	−1.8 ± 27.6	−13.8 ± 23.9	0.107
Swelling of the face (%)			
Baseline	52.0 ± 24.8	51.3 ± 24.1	0.925
5 weeks	44.0 ± 23.3	47.9 ± 23.8	0.559
8 weeks	48.6 ± 20.1	46.8 ± 22.0	0.762
Δ5	−8.0 ± 22.4	−3.4 ± 16.7	0.415
Δ8	−3.4 ± 22.1	−4.5 ± 25.0	0.863
Facial luster (%)			
Baseline	69.4 ± 14.7	67.0 ± 20.2	0.639
5 weeks	60.9 ± 17.7^#^	67.1 ± 16.1	0.204
8 weeks	59.2 ± 15.8^##^	61.3 ± 18.5	0.669
Δ5	−8.5 ± 16.1	0.0 ± 14.5	0.054
Δ8	−10.2 ± 14.4	−5.7 ± 18.7	0.350
Facial texture (%)			
Baseline	66.8 ± 17.8	74.2 ± 14.4	0.111
5 weeks	63.0 ± 19.8	67.8 ± 16.7^#^	0.366
8 weeks	58.2 ± 17.5^#^	66.2 ± 17.4^##^	0.113
Δ5	−3.8 ± 16.5	−6.4 ± 15.3	0.555
Δ8	−8.6 ± 14.6	−8.0 ± 13.7	0.885
Dullness of the face (%)			
Baseline	66.3 ± 23.4	73.3 ± 18.6	0.250
5 weeks	61.5 ± 20.9	67.9 ± 19.4	0.265
8 weeks	56.2 ± 20.9^##^	67.5 ± 18.8	0.048*
Δ5	−4.9 ± 11.4	−5.4 ± 13.5	0.886
Δ8	−10.2 ± 16.1	−5.7 ± 16.6	0.343
Blemishes on the face (%)			
Baseline	65.5 ± 18.5	64.7 ± 24.3	0.897
5 weeks	59.8 ± 20.1	64.9 ± 19.6	0.369
8 weeks	61.5 ± 21.8	61.7 ± 19.9	0.979
Δ5	−5.6 ± 15.7	0.3 ± 18.0	0.225
Δ8	−4.0 ± 11.1	−3.0 ± 18.1	0.824
Acne and pimples on the face (%)			
Baseline	38.3 ± 25.3	45.0 ± 28.0	0.378
5 weeks	39.3 ± 26.6	44.5 ± 28.6	0.506
8 weeks	47.0 ± 21.2	44.3 ± 29.4	0.702
Δ5	1.0 ± 20.6	−0.5 ± 29.3	0.836
Δ8	8.8 ± 21.4	−0.7 ± 25.7	0.162
Pores on nose and cheeks (%)			
Baseline	63.8 ± 20.4	72.1 ± 22.2	0.175
5 weeks	57.8 ± 23.3	68.8 ± 21.0	0.085
8 weeks	56.1 ± 24.9	69.0 ± 19.2	0.045*
Δ5	−6.0 ± 20.0	−3.3 ± 12.9	0.571
Δ8	−7.7 ± 23.5	−3.2 ± 11.3	0.381
Wrinkles around the eyes (%)			
Baseline	66.5 ± 18.0	59.5 ± 21.3	0.221
5 weeks	58.4 ± 20.6^#^	53.7 ± 26.7	0.493
8 weeks	58.8 ± 22.7^#^	58.3 ± 18.8	0.924
Δ5	−8.1 ± 12.8	−5.8 ± 16.8	0.594
Δ8	−7.7 ± 14.3	−1.3 ± 16.8	0.155
Dark circles under the eyes (%)			
Baseline	64.3 ± 22.8	62.7 ± 25.8	0.818
5 weeks	62.5 ± 21.5	56.8 ± 26.6	0.409
8 weeks	56.5 ± 24.0^#^	58.5 ± 25.2	0.775
Δ5	−1.8 ± 15.2	−6.0 ± 14.5	0.331
Δ8	−7.9 ± 17.3	−4.3 ± 24.1	0.549
Lip color (%)			
Baseline	62.5 ± 12.7	65.8 ± 17.8	0.450
5 weeks	55.0 ± 12.6^##^	62.1 ± 17.6	0.107
8 weeks	55.6 ± 12.3^##^	60.5 ± 17.5^#^	0.260
Δ5	−7.5 ± 9.6	−3.7 ± 11.1	0.201
Δ8	−6.8 ± 11.2	−5.3 ± 13.1	0.654
Have you suffered from psychological problems such as anxiety, depression, or irritability in the past month? (%)			
Baseline	36.8 ± 25.8	40.3 ± 22.6	0.599
5 weeks	35.8 ± 24.6	30.2 ± 21.4^#^	0.372
8 weeks	34.7 ± 23.6	33.1 ± 23.2	0.800
Δ5	−0.9 ± 15.0	−10.0 ± 21.5	0.076
Δ8	−2.1 ± 23.7	−7.2 ± 22.7	0.423
Have psychological factors interfered with your usual activities such as work, school, or housework in the past month? (%)			
Baseline	22.8 ± 25.0	21.9 ± 18.9	0.879
5 weeks	24.2 ± 23.5	25.4 ± 23.4	0.856
8 weeks	25.1 ± 24.1	23.5 ± 23.5	0.808
Δ5	1.4 ± 19.8	3.5 ± 22.3	0.719
Δ8	2.2 ± 23.1	1.6 ± 24.4	0.919

*Note:* Values are mean ± standard deviations. Delta values (Δ5 and Δ8) represent the degree of change at each intervention point relative to baseline. Visual analog scales (VAS): from none (minimum, 0%) to severe (maximum, 100%). *p* values were derived from comparisons between the postbiotic and placebo groups (intergroups) using an unpaired *t*‐test (*p < 0.05 vs. the placebo group). ^#^ *p* < 0.05, ^##^ *p* < 0.01, ^###^ *p* < 0.001 versus baseline within the same group using a Dunnett's test. Intragroup comparisons of delta values were not performed.

**TABLE 5:** Izumo scale scores for subjective assessment of gastrointestinal conditions.

Parameter	Postbiotic (*N* = 26)	Placebo (*N* = 28)	*p*
Heartburn			
Baseline	0 (0, 0)	0 (0, 0)	0.883
5 weeks	0 (0, 0.25)	0 (0, 1.0)	0.702
8 weeks	0 (0, 0)	0 (0, 0.75)	0.224
Stomach pain			
Baseline	0 (0, 0)	0 (0, 0)	0.306
5 weeks	0 (0, 0)	0 (0, 0.75)	0.490
8 weeks	0 (0, 0)	0 (0, 0)	0.279
Upset stomach			
Baseline	0 (0, 1.25)	0 (0, 1.0)	0.780
5 weeks	0 (0, 1.0)	0 (0, 2.0)	0.392
8 weeks	0 (0, 2.0)	0 (0, 2.0)	0.923
Constipation			
Baseline	2.0 (1.0, 4.0)	3.0 (2.0, 5.0)	0.080
5 weeks	1.0 (0, 3.0)	2.5 (1.0, 4.0)^#^	0.098
8 weeks	1.0 (0, 3.25)	2.5 (2.0, 4.0)^##^	0.112
Diarrhea			
Baseline	0 (0, 1.0)	0 (0, 1.75)	0.208
5 weeks	0 (0, 2.0)	0 (0, 1.0)^#^	0.858
8 weeks	0 (0, 0.25)	0 (0, 2.75)	0.194

*Note:* Median values are shown with the first and third interquartiles in parentheses. Each value for the five categories represents the sum of Izumo scale scores for 3 questions from no symptoms (minimum, grade 0) to severe (maximum, grade 5): heartburn, the sum of questions 1–3; stomach pain, the sum of questions 4–6; upset stomach, the sum of questions 7–9; constipation, the sum of questions 10–12; diarrhea. *p* values were derived from comparisons between the postbiotic and placebo groups (intergroups) using a Mann–Whitney *U*‐test (exact method version). ^#^ *p* < 0.05, ^##^ *p* < 0.01 versus baseline within the same group (intragroup) using the Wilcoxon signed rank exact test.

### 3.4 Safety

According to the judgment by a dermatologist, dryness, skin scaling, and papules on the face in both the postbiotic and placebo groups were improved after the intervention, and there was no significant difference between the two groups (Table 6). Erythema or scratch marks on the face were either absent or very mild at the start of the intervention and these parameters did not change significantly after the intervention. For hematological analysis, blood biochemical analysis, and urinalysis, some significant changes from baseline were observed for both groups (Table 7). These changes were small and the values remained within the normal range.

**TABLE 6:** Facial skin conditions in a safety assessment of the postbiotic formulation.

Parameter	Postbiotic (*N* = 30)	Placebo (*N* = 29 or 30)^a^	*p*
Dryness			
Baseline	3.0 (2.0, 3.0)	3.0 (2.0, 3.0)	0.792
5 weeks	2.0 (2.0, 3.0)^###^	2.0 (2.0, 3.0)^##^	0.472
8 weeks	2.0 (1.0, 2.0)^###^	2.0 (2.0, 3.0)^##^	0.161
Skin scaling			
Baseline	2.0 (1.0, 3.0)	2.0 (1.75, 2.0)	0.468
5 weeks	2.0 (1.0, 2.0)^##^	2.0 (1.0, 2.0)^#^	0.699
8 weeks	1.0 (1.0, 2.0)^###^	1.0 (1.0, 2.0)^##^	0.215
Erythema			
Baseline	1.0 (1.0, 1.0)	1.0 (1.0, 1.0)	1.000
5 weeks	1.0 (1.0, 1.0)	1.0 (1.0, 1.0)	0.304
8 weeks	1.0 (1.0, 1.0)	1.0 (1.0, 1.0)	0.655
Papules			
Baseline	2.0 (1.0, 2.0)	2.0 (1.0, 2.0)	0.816
5 weeks	2.0 (1.0, 2.0)	1.0 (1.0, 2.0)^###^	0.065
8 weeks	1.0 (1.0, 2.0)^##^	1.0 (1.0, 2.0)^#^	0.804
Scratch marks			
Baseline	1.0 (1.0, 1.0)	1.0 (1.0, 1.0)	0.154
5 weeks	1.0 (1.0, 1.0)	1.0 (1.0, 1.0)	1.000
8 weeks	1.0 (1.0, 1.0)	1.0 (1.0, 1.0)	0.309

*Note:* Median values are shown with the first and third interquartiles in parentheses. Each value of the five categories is judged from no symptoms (minimum, grade 1) to severe condition (maximum, grade 5). Before and after the intervention, no subject in either group was assessed as grade 4 or 5. *p* values were derived from comparisons between the postbiotic and placebo groups using a Mann–Whitney *U*‐test (exact method version). ^#^ *p* < 0.05, ^##^ *p* < 0.01 and ^###^ *p* < 0.001 versus baseline within the same group using a Wilcoxon signed rank exact test.

^a^ The placebo group had 30 participants at study initiation and 29 participants completed the intervention period.

**TABLE 7:** Physical and biochemical parameters for the full analysis set (FAS).

Parameter		Postbiotic (*N* = 30)	Placebo (*N* = 29 or 30)^a^	*p*
Physical parameters				
Body weight (kg)	Pre	54.2 ± 11.1	51.0 ± 7.2	0.199
	Post	54.8 ± 11.5^*##*^	51.5 ± 7.3^#^	0.193
Blood pressures (mmHg)				
Systolic	Pre	109.7 ± 14.6	107.1 ± 12.6	0.462
	Post	112.0 ± 13.8	111.6 ± 13.9^#^	0.902
Diastolic	Pre	69.7 ± 12.4	68.6 ± 7.5	0.661
	Post	70.8 ± 10.2	73.1 ± 11.4^*##*^	0.417
Hematologic parameters				
White blood cells (/μL)	Pre	6097 ± 1524	6213 ± 1645	0.777
	Post	5880 ± 1503	5910 ± 1326	0.935
Red blood cells (×10^4^/μL)	Pre	433.6 ± 32.8	435.1 ± 23.0	0.838
	Post	421.8 ± 33.2^#^	437.5 ± 27.1	0.052
Hemoglobin (g/dL)	Pre	13.1 ± 0.9	12.9 ± 0.8	0.325
	Post	12.6 ± 1.0^###^	12.8 ± 1.0	0.416
Hematocrit (%)	Pre	40.8 ± 2.8	40.1 ± 2.4	0.324
	Post	39.8 ± 2.9^#^	40.6 ± 2.6	0.256
Platelet (×10^4^/μL)	Pre	28.9 ± 7.5	27.3 ± 5.2	0.340
	Post	28.8 ± 6.9	27.6 ± 5.4	0.489
MCV (fL)	Pre	94.1 ± 5.1	92.2 ± 4.3	0.112
	Post	94.5 ± 5.0	92.9 ± 4.5^*##*^	0.199
MCH (pg)	Pre	30.3 ± 2.0	29.6 ± 1.6	0.171
	Post	30.0 ± 2.1^#^	29.3 ± 1.9^*##*^	0.243
MCHC (%)	Pre	32.2 ± 0.8	32.2 ± 0.8	0.961
	Post	31.7 ± 1.1^*##*^	31.6 ± 1.0^###^	0.594
Blood biochemical parameters				
Total protein (g/dL)	Pre	7.3 ± 0.4	7.2 ± 0.3	0.837
	Post	7.1 ± 0.5^#^	7.2 ± 0.4	0.432
Albumin (g/dL)	Pre	4.4 ± 0.2	4.4 ± 0.2	0.380
	Post	4.3 ± 0.3^#^	4.5 ± 0.3	0.039*
AST (U/L)	Pre	18.3 ± 4.8	17.9 ± 4.1	0.708
	Post	16.8 ± 4.5^#^	18.1 ± 5.4	0.302
ALT (U/L)	Pre	13.9 ± 6.4	14.5 ± 6.3	0.730
	Post	13.7 ± 5.6	15.3 ± 9.7	0.445
LDH (U/L)	Pre	166.2 ± 26.3	158.1 ± 25.4	0.230
	Post	158.6 ± 26.9^#^	151.7 ± 27.8^#^	0.334
Total bilirubin (mg/dL)	Pre	0.7 ± 0.3	0.7 ± 0.2	0.956
	Post	0.7 ± 0.2^#^	0.7 ± 0.2	0.694
ALP (U/L)	Pre	56.3 ± 14.5	58.9 ± 14.5	0.486
	Post	54.4 ± 12.9	57.3 ± 13.5	0.400
γ‐GPT (U/L)	Pre	18.5 ± 12.7	14.8 ± 5.0	0.149
	Post	17.0 ± 11.3	14.1 ± 4.7	0.198
Urea nitrogen (mg/dL)	Pre	11.9 ± 2.4	12.9 ± 3.9	0.247
	Post	12.5 ± 2.5	12.4 ± 3.2	0.842
Creatinine (mg/dL)	Pre	0.61 ± 0.09	0.58 ± 0.07	0.083
	Post	0.63 ± 0.09	0.59 ± 0.09	0.113
Uric acid (mg/dL)	Pre	4.2 ± 0.7	3.9 ± 0.9	0.169
	Post	4.2 ± 0.7	4.0 ± 1.1	0.377
Sodium (mEq/L)	Pre	140.7 ± 2.0	139.9 ± 1.5	0.078
	Post	141.1 ± 1.7	140.8 ± 2.0^#^	0.435
Chloride (mEq/L)	Pre	104.1 ± 2.0	103.6 ± 1.7	0.296
	Post	105.0 ± 1.8^#^	104.3 ± 1.6	0.098
Potassium (mEq/L)	Pre	4.3 ± 0.3	4.3 ± 0.4	0.819
	Post	4.0 ± 0.3^###^	4.0 ± 0.3^###^	0.865
Calcium (mg/dL)	Pre	9.4 ± 0.3	9.4 ± 0.3	0.800
	Post	9.3 ± 0.3	9.4 ± 0.3	0.440
Total‐Cho (mg/dL)	Pre	205.7 ± 36.2	201.6 ± 36.1	0.660
	Post	196.1 ± 31.7^##^	198.2 ± 34.2	0.804
LDL‐Cho (mg/dL)	Pre	114.7 ± 28.6	112.1 ± 27.9	0.726
	Post	107.7 ± 26.9^##^	109.6 ± 25.4	0.779
HDL‐Cho (mg/dL)	Pre	78.3 ± 21.7	76.8 ± 17.3	0.768
	Post	75.9 ± 19.1	74.8 ± 17.5	0.823
Triglyceride (mg/dL)	Pre	62.9 ± 34.2	66.8 ± 35.0	0.662
	Post	63.4 ± 29.1	72.3 ± 38.6	0.319
Fasting glucose (mg/dL)	Pre	84.1 ± 8.7	81.0 ± 5.3	0.104
	Post	85.1 ± 11.2	83.0 ± 8.4	0.427
Hemoglobin A1c (%)	Pre	5.2 ± 0.3	5.2 ± 0.2	0.919
	Post	5.3 ± 0.2^#^	5.2 ± 0.3	0.720
Urine parameters				
pH	Pre	5.9 ± 0.4	6.1 ± 0.6	0.110
	Post	6.2 ± 0.5^#^	6.3 ± 0.6	0.346
Gravity	Pre	1.023 ± 0.006	1.020 ± 0.007	0.177
	Post	1.020 ± 0.006	1.020 ± 0.006	0.999
Protein, qualitative	Pre	0 (0, 0)	0 (0, 0)	0.690
	Post	0 (0, 0) ^#^	0 (0, 0)	0.147
Glucose, qualitative	Pre	0 (0, 0)	0 (0, 0)	1.000
	Post	0 (0, 0)	0 (0, 0)	1.000
Urobilinogen, qualitative	Pre	0 (0, 0)	0 (0, 0)	0.317
	Post	0 (0, 0)	0 (0, 0)	0.309
Bilirubin, qualitative	Pre	0 (0, 0)	0 (0, 0)	1.000
	Post	0 (0, 0)	0 (0, 0)	1.000
Ketone, qualitative	Pre	0 (0, 0)	0 (0, 0)	0.317
	Post	0 (0, 0)	0 (0, 0)	1.000
Blood, qualitative	Pre	0 (0, 0)	0 (0, 0.25)	0.357
	Post	0 (0, 0)	0 (0, 0)	0.493

*Note:* Values are mean ± standard deviations or median with the first and third interquartiles in parentheses. *p* values were derived from comparisons between the postbiotic and placebo groups using an unpaired Student's or Welch's *t*‐test (for quantitative parameters) or Mann–Whitney *U*‐test (exact method version, for qualitative parameters) (*p < 0.05). ^#^ *p* < 0.05, ^##^ *p* < 0.01 and ^###^ *p* < 0.001 versus pre‐intervention within the same group using Dunnett's test (quantitative parameters) or Wilcoxon signed rank exact test (qualitative parameters).

Abbreviations: ALP, alkaline phosphatase; ALT, alanine aminotransferase; AST, aspartate aminotransferase; Cho, cholesterol; LDH, lactate dehydrogenase; MCH, mean corpuscular hemoglobin; MCHC, mean corpuscular hemoglobin‐concentration; MCV, mean corpuscular volume; γ‐GPT, γ‐glutamic transpeptidase.

^a^ The placebo group had 30 participants at study initiation and 29 participants completed the intervention period.

Adverse events reported for each group were mild/moderate and transient and resolved within a few days. One subject in the postbiotic group developed a *Herpes labialis* infection on the lip and recovered after 8 days. Other adverse events included menstruation‐related pain, gastrointestinal pain caused by food poisoning, neck/lower back pain, and stiff shoulders or tired/dry eyes, all of which were judged by the investigator to be clinically irrelevant and unrelated to the intervention. There were no significant differences in the frequency of adverse events between the postbiotic and placebo groups (*p* = 0.195, Fisher's exact test).

### 3.5 Sub‐Analyses on Participants in Their 40s

**FIGURE 3:**
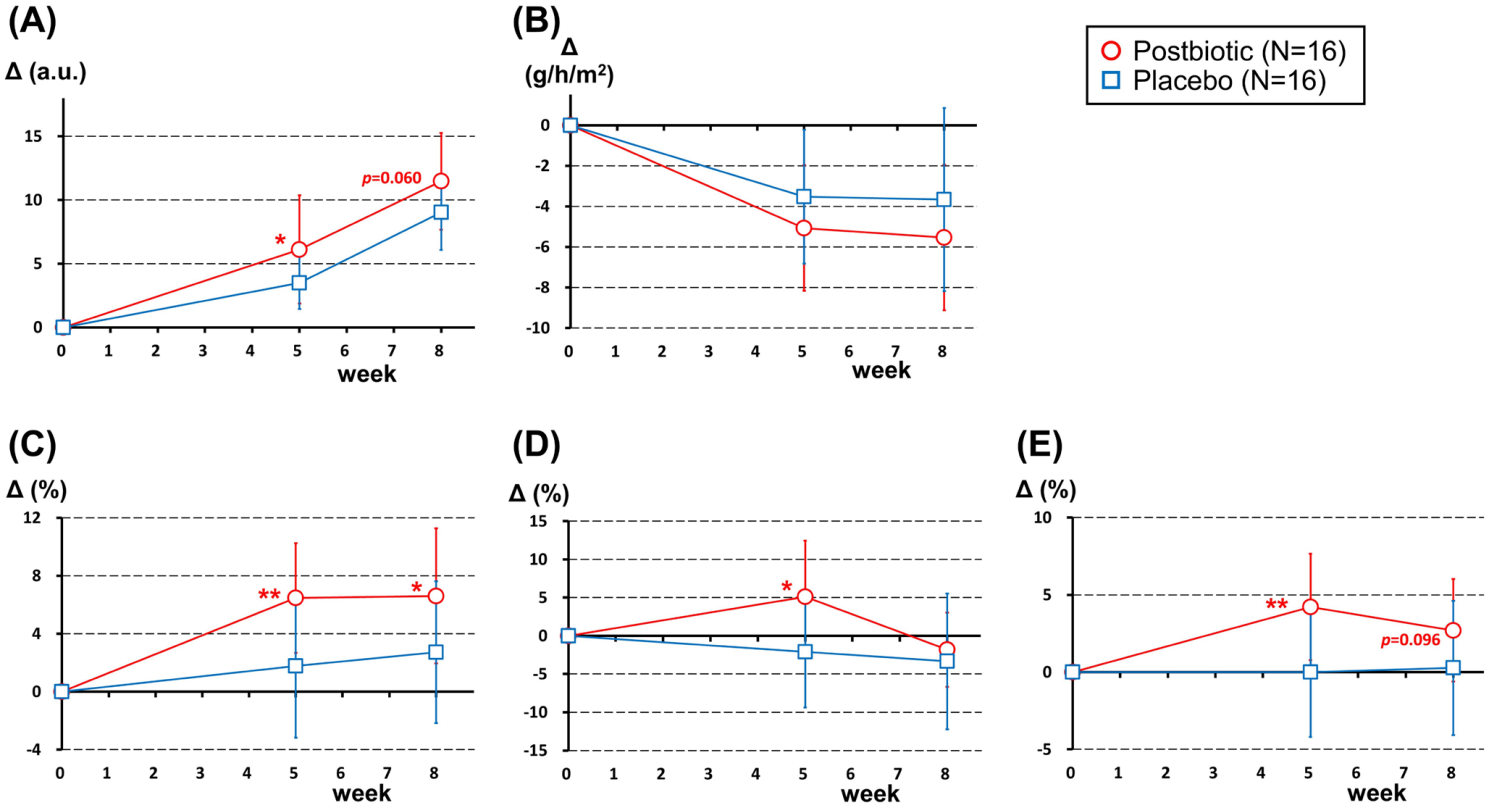
Effect of postbiotics on changes in the three primary endpoints in a stratified analysis of participants in their 40s. Data for groups given the postbiotic and placebo are shown in red and blue, respectively. Open symbols correspond to mean values and error bars indicate standard deviations. Delta values at 5 and 8 weeks after intervention are represented as the change in value relative to the baseline (week 0). (A) Stratum corneum water content, (B) Transepidermal water loss (TEWL), (C–E) Elasticity. (C) Skin recovery rate (R2); (D) net elasticity (R5); (E) skin firmness (R7). *p < 0.05, **p < 0.01 and other *p* values were derived from between‐group comparisons.

Objective data for skin in subjects in their 40s (the mean ages in the postbiotic and placebo groups were 45.6 and 45.3 years, respectively; Table 8) were gathered and evaluated as primary endpoints. The stratum corneum water content on the cheek in the postbiotic and placebo groups increased after the intervention (Table 8), but the change between weeks 0 to 5 seen for the postbiotic group was significantly greater (*p* = 0.043) and the change between weeks 0 to 8 also tended to be greater (*p* = 0.060) compared with that for the placebo group (Figure 3A). TEWL decreased significantly after the intervention for both groups (Table 8), and although the skin condition was improved, there was no significant difference between the groups in terms of the amount of change (Figure 3B). Participants in the postbiotic supplementation group had significantly increased R2 and R7 for skin elasticity (skin recovery rate and skin firmness, respectively) at both 5 and 8 weeks after the intervention. R5 (net skin elasticity) was also increased significantly at the 5‐week point. On the other hand, placebo supplementation did not affect these parameters (Table 8). The postbiotic group also showed significantly greater changes in all three elasticity parameters compared to the placebo group (Figure 3C–E). We next analyzed the VAS scores for facial condition of participants in their 40s (Table 9). Skin itchiness decreased significantly from baseline for the placebo group, but not the postbiotic group, likely due to the low baseline levels, meaning the symptoms in the postbiotic group may have been slight or mild. The appearance of pores on the nose and cheeks in the postbiotic group was significantly less than in the placebo group at 5 weeks after intervention (*p* = 0.044) and tended to be significantly less at week 8 (*p* = 0.081). Facial luster and the appearance of wrinkles around the eyes, dark circles under the eyes, and lip color seemed to reduce in part only for the postbiotic group and not the placebo group.

**TABLE 8:** Objective evaluation of cheek skin parameters for participants in their 40s.

Parameter	Postbiotic (*N* = 16)	Placebo (*N* = 16)	*p*
Age (year)			
Baseline	45.6 ± 2.81	45.3 ± 3.20	0.821
Stratum corneum water content (a.u.)			
Baseline	33.1 ± 7.9	33.1 ± 6.6	0.981
5 weeks	39.2 ± 9.0^###^	36.6 ± 7.4^###^	0.387
8 weeks	44.6 ± 6.8^###^	42.2 ± 7.8^###^	0.367
TEWL (g/h/m^2^)			
Baseline	20.0 ± 4.5	21.0 ± 7.4	0.666
5 weeks	14.9 ± 3.1^###^	17.4 ± 4.9^##^	0.100
8 weeks	14.5 ± 3.2^###^	17.3 ± 6.3^###^	0.121
Elasticity (%)			
R2			
Baseline	56.7 ± 9.1	59.9 ± 7.5	0.285
5 weeks	63.1 ± 8.0^###^	61.7 ± 7.0	0.584
8 weeks	63.3 ± 10.8^###^	62.6 ± 7.8	0.844
R5			
Baseline	52.3 ± 8.0	55.1 ± 9.7	0.384
5 weeks	57.5 ± 7.9^##^	53.0 ± 7.4	0.111
8 weeks	50.5 ± 6.3	51.8 ± 8.2	0.628
R7			
Baseline	34.3 ± 6.5	36.6 ± 6.0	0.310
5 weeks	38.6 ± 6.1^###^	36.6 ± 5.7	0.362
8 weeks	37.0 ± 6.9^#^	36.9 ± 6.6	0.954

*Note:* Values are mean ± standard deviations. *p* values were derived from comparisons between the postbiotic and placebo groups (intergroups) using an unpaired *t*‐test. ^#^ *p* < 0.05, ^##^ *p* < 0.01, ^###^ *p* < 0.001 versus baseline within the same group using Dunnett's test.

Abbreviations: a.u., arbitrary unit; R2, skin recovery rate (total recovery amplitude/maximum amplitude by suction, Ua/Uf); R5, net elasticity (immediate refractive, Ur/Ue); R7, skin firmness (immediate recovery after relax/maximum amplitude by suction, Ur/Uf); TEWL, transepidermal water loss.

**TABLE 9:** Subjective assessment of cheek skin parameters for participants in their 40s.

Parameter	Postbiotic (*N* = 16)	Placebo (*N* = 16)	*p*
Skin dryness (%)			
Baseline	72.5 ± 10.4	72.6 ± 19.6	0.982
5 weeks	51.1 ± 17.0^###^	55.4 ± 20.6^###^	0.524
8 weeks	40.6 ± 21.3^###^	53.9 ± 19.0^###^	0.072
Δ5	−21.4 ± 19.3	−17.2 ± 17.3	0.524
Δ8	−31.9 ± 22.6	−18.7 ± 18.0	0.078
Skin elasticity (%)			
Baseline	74.6 ± 11.9	76.3 ± 14.2	0.718
5 weeks	56.7 ± 15.2^###^	59.6 ± 17.4^###^	0.615
8 weeks	57.9 ± 15.5^###^	62.7 ± 16.8^##^	0.405
Δ5	−17.9 ± 18.1	−16.6 ± 15.7	0.836
Δ8	−16.7 ± 17.5	−13.6 ± 17.5	0.617
Skin itchiness (%)			
Baseline	22.1 ± 24.0	35.6 ± 29.5	0.167
5 weeks	17.6 ± 17.8	20.9 ± 18.0^*#*^	0.604
8 weeks	19.3 ± 20.9	23.1 ± 23.0^*#*^	0.627
Δ5	−4.5 ± 20.8	−14.7 ± 22.7	0.196
Δ8	−2.9 ± 24.7	−12.6 ± 24.1	0.270
Facial swelling (%)			
Baseline	50.6 ± 25.7	52.1 ± 21.2	0.858
5 weeks	44.4 ± 22.5	47.3 ± 22.4	0.720
8 weeks	46.6 ± 19.6	48.8 ± 23.3	0.782
Δ5	−6.2 ± 24.5	−4.8 ± 17.4	0.856
Δ8	−4.0 ± 24.5	−3.4 ± 25.5	0.944
Facial luster (%)			
Baseline	69.5 ± 15.8	64.1 ± 22.4	0.438
5 weeks	59.9 ± 19.3	64.9 ± 16.8	0.446
8 weeks	57.6 ± 14.2^*#*^	58.9 ± 17.1	0.815
Δ5	−9.6 ± 18.8	0.8 ± 16.2	0.107
Δ8	−11.9 ± 15.6	−5.2 ± 20.5	0.308
Facial texture (%)			
Baseline	67.8 ± 18.0	72.3 ± 15.8	0.458
5 weeks	59.9 ± 21.6	65.4 ± 15.9	0.418
8 weeks	58.6 ± 14.1	65.4 ± 18.0	0.247
Δ5	−7.9 ± 16.1	−6.9 ± 12.7	0.847
Δ8	−9.1 ± 15.0	−6.9 ± 12.9	0.652
Dullness of the face (%)			
Baseline	65.5 ± 27.5	74.8 ± 15.8	0.250
5 weeks	59.6 ± 24.1	68.2 ± 17.1	0.252
8 weeks	52.5 ± 23.7^##^	65.6 ± 18.9^*#*^	0.094
Δ5	−5.9 ± 13.1	−6.6 ± 12.2	0.879
Δ8	−13.0 ± 17.6	−9.2 ± 13.0	0.492
Blemishes on the face (%)			
Baseline	67.0 ± 19.6	63.5 ± 29.2	0.693
5 weeks	63.4 ± 20.2	67.2 ± 20.1	0.596
8 weeks	63.3 ± 24.1	61.1 ± 21.1	0.781
Δ5	−3.6 ± 16.4	3.7 ± 16.9	0.223
Δ8	−3.7 ± 12.0	−2.4 ± 19.6	0.829
Acne and pimples on the face (%)			
Baseline	33.8 ± 23.6	36.6 ± 27.3	0.752
5 weeks	33.3 ± 25.4	41.5 ± 28.9	0.402
8 weeks	42.4 ± 18.2	40.6 ± 30.3	0.839
Δ5	−0.4 ± 20.2	4.9 ± 28.1	0.544
Δ8	8.6 ± 23.5	3.9 ± 22.5	0.569
Pores on the nose and cheeks (%)			
Baseline	61.9 ± 21.9	70.4 ± 21.6	0.275
5 weeks	54.2 ± 25.3	70.1 ± 16.3	0.044*
8 weeks	52.9 ± 25.5	67.0 ± 17.8	0.081
Δ5	−7.7 ± 20.3	−0.4 ± 10.8	0.214
Δ8	−8.9 ± 26.6	−3.4 ± 11.7	0.455
Wrinkles around the eyes (%)			
Baseline	67.1 ± 21.3	66.3 ± 17.0	0.906
5 weeks	57.4 ± 23.2^*#*^	60.8 ± 21.9	0.676
8 weeks	57.8 ± 25.1^*#*^	58.6 ± 18.7	0.924
Δ5	−9.7 ± 13.7	−5.5 ± 15.1	0.419
Δ8	−9.3 ± 13.4	−7.8 ± 13.2	0.742
Dark circles under the eyes (%)			
Baseline	67.4 ± 23.9	66.0 ± 22.7	0.869
5 weeks	64.8 ± 21.1	59.6 ± 23.5	0.516
8 weeks	56.9 ± 24.5^*#*^	57.9 ± 25.2	0.910
Δ5	−2.6 ± 14.2	−6.4 ± 14.1	0.453
Δ8	−10.5 ± 14.1	−8.1 ± 25.4	0.746
Lip color (%)			
Baseline	62.7 ± 14.1	62.4 ± 17.9	0.965
5 weeks	52.8 ± 13.3^##^	60.9 ± 17.6	0.149
8 weeks	53.1 ± 11.8^##^	58.3 ± 15.4	0.293
Δ5	−9.9 ± 10.4	−1.5 ± 9.5	0.023*
Δ8	−9.6 ± 9.6	−4.1 ± 11.5	0.156

*Note:* Values are mean ± standard deviations. Delta values (Δ5 and Δ8) represent the change from baseline at each intervention timepoint. Visual analog scales (VAS): from none (minimum, 0%) to severe (maximum, 100%). *p* values were derived from comparisons between the postbiotic and placebo groups (intergroups) using an unpaired *t*‐test (*p < 0.05 vs. the placebo group). ^#^ *p* < 0.05, ^##^ *p* < 0.01, ^###^ *p* < 0.001 versus baseline within the same group using Dunnett's test. Intragroup comparisons of delta values were not performed.

## 4 Discussion

This study demonstrated that our postbiotic supplement with *L. sakei* KABP‐065 may improve cheek skin condition in healthy Japanese women in their 30s and 40s. In a stratified analysis to consider only participants in their 40s, this postbiotic supplementation not only helped improve facial elasticity but also helped maintain cheek moisture. These findings are based on scientifically accurate evaluation methods using specialized instruments, rather than on subjective evaluations, which can be influenced by the study participant's mood at the time of the test. On the other hand, women generally have many opportunities to touch and observe their own skin, especially skin on their face, so they can be expected to be sensitive to and be able to judge slight changes in the condition of their skin. Thus, some subjective evaluations on the face can also be valuable. In fact, the results showed that participants in the postbiotic group were more likely to show improvements in facial luster, dullness of the face, pores on the nose and cheeks, and wrinkles around the eyes than the placebo group. We also confirmed the absence of gastrointestinal or psychological issues before beginning the intervention in this study and none of these parameters worsened after supplementation with *L. sakei* KABP‐065 for 8 weeks. Furthermore, our supplement included heat‐killed bacteria that allow more accurate and continuous intake of the required number of bacteria than use of live probiotics. Continuous supplementation with heat‐killed bacteria is thus more manageable and safer for study participants and also can have more consistent biogenic effects.

The etiology of AD is multifactorial and involves complex interactions of genetic, environmental, and immunological responses [31, 32, 33]. Up to 90% of AD patients experience *Staphylococcus aureus* infection, and colonization with *S. aureus* is associated with AD severity [34, 35, 36]. Some studies using an AD‐prone model mouse showed that oral administration of *L. sakei* KABP‐065 significantly reduces AD severity [37], has a strong inhibitory effect against *S. aureus* , and reduces serum IgE production [20]. Furthermore, *L. sakei* KABP‐065 was shown to improve skin barrier function in clinical trials involving children and adults with AD [21, 26, 38]. The possible mechanism responsible for this improvement in skin barrier function is the ability of *L. sakei* KABP‐065 to suppress excessive activities of thymus and activation‐regulated chemokine (TARC) that regulates immune system function. The enhancement of barrier function can protect the skin against external stimuli such as antigens and physical stimuli to help restore metabolic function in the skin to healthy levels [21, 27, 39]. In the present study, the participants were healthy and expected to have normal immune system function, meaning that they likely do not have immune system hyperactivity often seen in AD. Therefore, the effects seen in this study may be weaker than those that might be obtained for patients with AD. On the other hand, the results of the present study suggested that *L. sakei* KABP‐065 may more positively affect the skin of women in their 40s than in the PPS population that included women in their 30s. Japanese women often begin to experience disturbances and decreases in estrogen levels in their mid‐ to late‐40s. In our previous clinical study involving Japanese women, which included women who were older (mean age, 49.95 years old) than the average age of subjects in the present study (in their 40s), we demonstrated that serum estrone and estrogen levels in the placebo group decreased significantly after 12 weeks of intervention [40]. As described in the Introduction, skin aging can be caused by external and internal factors [1]. For women, changes in estrogen regulatory mechanisms (estrobolome) occurring during menopause could also affect skin structure [41, 42, 43]. Thus, we expect that further studies evaluating the relationship between estrobolome and effects on the skin will help elucidate novel mechanism(s) by which *L. sakei* KABP‐065 improves the skin condition of healthy middle‐aged women.

The test conditions in the study appeared to have some limitations for obtaining a clear demonstration of the effects of *L. sakei* KABP‐065 on healthy skin conditions. First, in healthy participants, changes in parameters could be smaller than those seen in previous clinical trials involving patients with AD.

Second, many study participants may have experienced behavioral changes during the intervention period. This study coincided with the emergence of COVID‐19 and a period when the government of Japan expected Japanese citizens to modify their behavior to avoid outdoor activities and socialization to reduce the risk of COVID‐19 infection. However, behavioral restrictions set forth by the government during COVID‐19 were not mandatory, and there was a movement to voluntarily lift behavioral restrictions in Japan. From late autumn in 2022 to early spring in 2023, nearly all outdoor amusement parks, zoos, and other outdoor recreation sites that had been closed during COVID‐19 resumed operations, while schools and workplaces encouraged resumption of on‐site activities. In fact, we experienced serious compliance violations by some participants that affected the quality of the study, such as visiting outdoor facilities or actively participating in outdoor recreation.

Another factor that could have complicated evaluation of the effects of the KABP‐065 postbiotic was that face masks and/or protective glasses worn during the study period could have protected facial skin from normal external stimuli. The present study was conducted during the season when cedar and cypress pollen typically disperse, but during the intervention period the pollen levels in Tokyo, Japan, increased earlier and to much higher (> 1.8‐fold) levels [44]. To avoid this increased pollen, and also to prevent COVID‐19 infection, many people strictly followed preventive methods involving masking. Wearing of masks or protective glasses may themselves have improved many skin‐related parameters in the placebo group, making the effectiveness of postbiotics less visible. Thus, additional trials conducted in areas with low pollen dispersion and during a period when masking is less common could clarify the beneficial effects of *L. sakei* KABP‐065 supplementation in healthy people.

The scale of this study was small, and each parameter was evaluated using standard statistical methods commonly used in clinical trials of similar size. The efficacy of this postbiotic may be more accurately evaluated using more advanced methods appropriate for the target population. We therefore focused on evaluation of TEWL and employed a linear mixed‐effects model (LMM) analysis for repeated measures with baseline, treatment, and study visit (at 5‐ or 8‐week) as factors and an unstructured covariance matrix. In the LMM, we noted a significant difference between the groups after the intervention (*p* = 0.021) but not time (between 5 and 8 weeks after intervention). After adjusting for the baseline, the TEWL was approximately 1.5 g/h/m^2^ lower for the postbiotic group than for the placebo group at weeks 5 and 8 (means ± standard errors; 15.44 ± 0.48 at the 5‐week timepoint and 15.42 ± 0.61 at the 8‐week timepoint for the postbiotic group, 17.00 ± 0.46 at the 5‐week timepoint and 16.98 ± 0.59 at the 8‐week timepoint for the placebo group). These results suggest that for healthy women the KABP‐065 postbiotic may have a positive effect on all three primary endpoints (skin dryness, skin elasticity, and TEWL). This possibility should be confirmed in larger and more complex trials involving more participants as well as a study cohort that has a broader range of age and severity of skin issues. These trials could also test different doses and intervention periods for postbiotic supplementation that could provide greater insight into the most effective, safe, and sustainable ways to help people improve skin conditions.

In conclusion, here we demonstrated that heat‐treated *L. sakei* KABP‐065 supplementation may improve facial skin conditions in healthy women in their 30s and 40s. These findings can provide a basis for future studies to evaluate symptom improvement of skin conditions in middle‐aged women.

## Author Contributions

Jinko Sawashita: project administration (lead); conceptualization (equal); methodology (equal); investigation (equal); validation (equal); formal analysis (equal); visualization (lead); writing – original draft (lead); writing – review and editing (lead). Shinichi Honda: conceptualization (equal); methodology (equal); investigation (equal); validation (equal); formal analysis (equal); visualization (supporting); writing – original draft (supporting); writing – review and editing (supporting). Yuji Tominaga: conceptualization (equal); methodology (equal); investigation (equal); validation (equal); formal analysis (equal); writing – review and editing (supporting). Namiko Tomatsu; investigation (supporting); formal analysis (supporting); writing – review and editing (supporting). Jordi Espadaler‐Mazo: conceptualization (equal); validation (equal); formal analysis (equal); writing – original draft (supporting). Takahiro Ueda: supervision (lead); conceptualization (equal); methodology (equal); investigation (supporting).

## Funding

This study was funded by the Kaneka Corporation.

## Disclosure

Declaration: The experiments in this study complied with the current laws of the country in which they were performed.

## Ethics Statement

The protocol for this clinical study was approved by the ethics committees of the Clinical Research Review Center (Tokyo, Japan; January 11, 2023; approval No. CrrC23‐02). This study was also registered at the University Hospital Medical Information Network Clinical Trials Registry in Japan (registration No. UMIN000050034). All volunteers provided written informed consent to participate. The study was performed in accordance with the Declaration of Helsinki (adopted in 1964 and revised in October 2013), Ethical Guidelines for Medical and Health Research Involving Human Subjects (Notification No. 3 issued in 2014 by the Ministry of Education, Culture, Sports, Science and Technology and the Ministry of Health, Labor and Welfare in Japan), and the Act on the Protection of Personal Information (Act No. 57 issued May 30, 2003 in Japan).

## Conflicts of Interest

All authors are employees of Kaneka Corporation or AB‐Biotics S.A. as a partner company of Kaneka Corporation and declare no conflicts of interest. None of the co‐authors would receive financial benefits from the commercial development of the postbiotic formulation.

## Data Availability

Data sharing not applicable to this article as no datasets were generated or analyzed during the current study.
